# High-throughput quantification of ochronotic pigment formation in *Escherichia coli* to evaluate the potency of human 4-hydroxyphenylpyruvate dioxygenase inhibitors in multi-well format

**DOI:** 10.1016/j.mex.2020.101181

**Published:** 2020-12-13

**Authors:** Jessie Neuckermans, Sien Lequeue, Alan Mertens, Steven Branson, Ulrich Schwaneberg, Joery De Kock

**Affiliations:** a Department of In Vitro Toxicology and Dermato-Cosmetology, Faculty of Medicine and Pharmacy, Vrije Universiteit Brussel, Laarbeeklaan 103, 1090 Brussels, Belgium; b Lehrstuhl für Biotechnologie, RWTH Aachen University, Worringerweg 3, 52074 Aachen, Germany

**Keywords:** High-throughput screening, In Vitro, Bacterial cell culture, Colorimetric, Inhibitor testing, Tyrosinemia, Ochronotic pigment, 4-Hydroxyphenylpyruvate dioxygenase

## Abstract

4-hydroxyphenylpyruvate dioxygenase (HPD) is a key enzyme in the catabolism of tyrosine and therefore of great importance as a drug target to treat tyrosine-related inherited metabolic disorders (TIMD). Inhibition of this enzyme is therapeutically applied to prevent accumulation of toxic metabolites in TIMD patients. Nowadays an ex-herbicide, nitisinone, is used for this purpose and many more inhibitors are being explored and need to be tested. Here, we describe a colorimetric bacterial whole-cell screening system that allows quantifying the inhibitory effects of new human HPD inhibitors in a high-throughput and robust fashion. For this high-throughput screening (HTS) system we rely on the capability of recombinant *E. coli* that express human HPD, to generate a brown ochronotic pigment after the addition of tyrosine, whereafter this brown pigment can be quantified in a very specific and sensitive way by spectrophotometric analysis. Altogether, this robust and simple HTS screening system can be described as non-harmful, non-laborious and cost-effective with the aim to identify and evaluate novel therapeutic human HPD inhibitors for the treatment of TIMD.

• This robust high-throughput screening system enables rapid identification and evaluation of potential inhibitors of human 4-hydroxyphenylpyruvate dioxygenase.
• Simple and fast colorimetric quantification of the formation of ochronotic pigment.

## Graphical abstract

**Figure:**
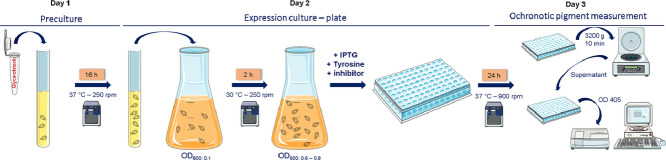

Specifications table

**Table:** 

Subject Area:	Pharmacology, Toxicology and Pharmaceutical Science
More specific subject area:	Preclinical drug testing
Method name:	High-throughput quantification of ochronotic pigment formation in *Escherichia coli*.
Name and reference of original method:	Neuckermans, J., Mertens, A., De Win, D. *et al.* A robust bacterial assay for high-throughput screening of human 4-hydroxyphenylpyruvate dioxygenase inhibitors. *Sci Rep***9,** 14145 (2019).
Resource availability:	N/A

## Method details

**Fig. 1:**
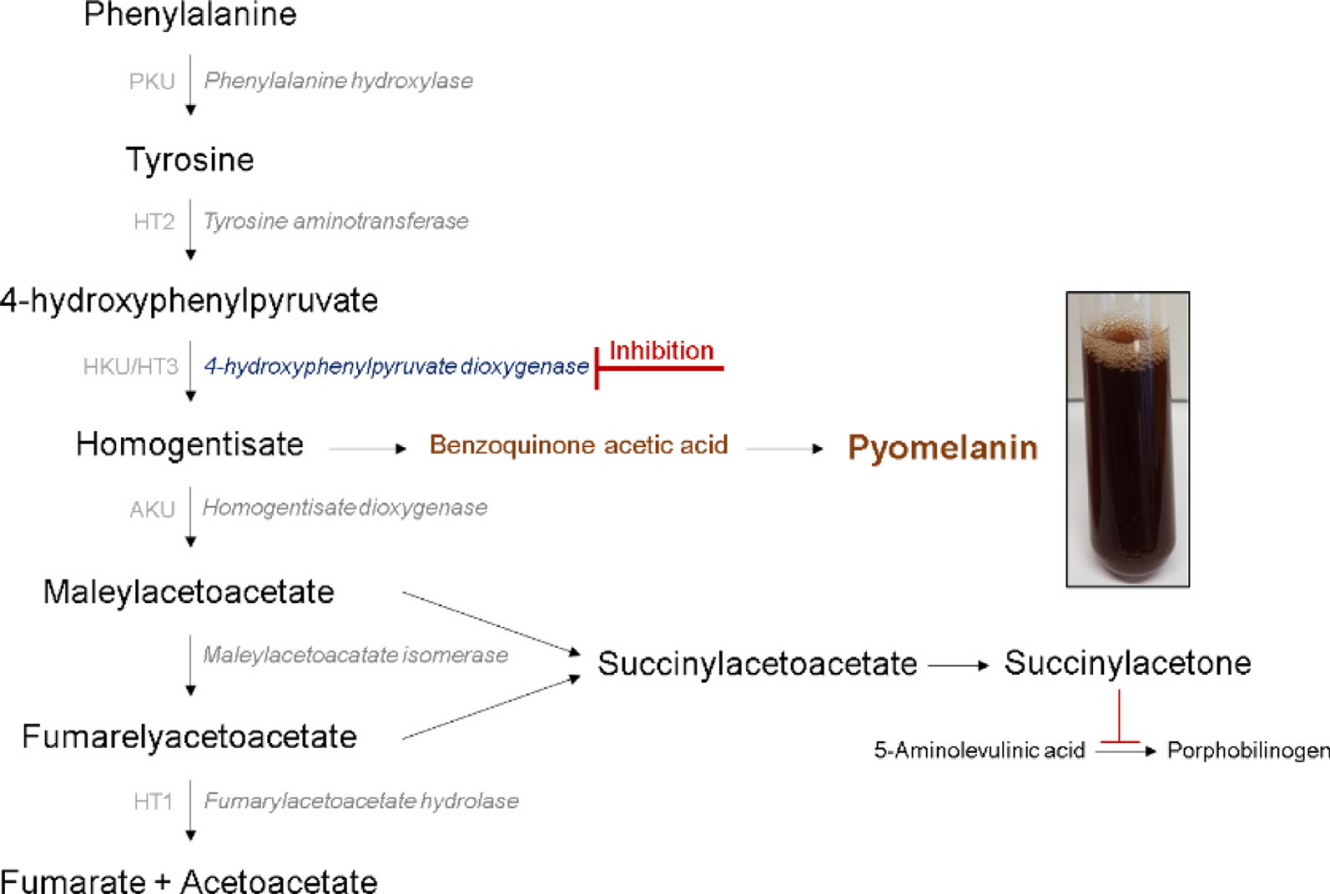
**Tyrosine degradation pathway** comprises a 5-step enzymatic conversion from tyrosine to fumarate and acetoacetate. Tyrosine derives from the diet or hydroxylation of phenylalanine by phenylalanine hydroxylase and is converted to 4-hydroxyphenylpyruvate (HPP) by tyrosine aminotransferase, the first step in the tyrosine catabolism. Further, 4-HPD catalyzes the formation of homogentisate (HGA) out of HPP, which is converted to maleylacetoacetate (MAA) by homogentisate dioxygenase. Excess of HGA can accumulate, auto-oxidize and polymerize to an ochronotic pigment, i.e. pyomelanin. The penultimate step comprises the conversion of MAA to fumarylacetoacetate (FAA) by maleylacetoacetate isomerase. Lastly, FAA will be broken down to fumarate and acetoacetate by the final enzyme fumarylacetoacetate hydrolase (FAH). When FAH functionality is deficient, MAA and FAA can also be converted into succinylacetone which inhibits the porphobilinogen synthesis. 5 different TIMD are related to an inefficient or deficient enzyme in this pathway.

The human enzyme 4-hydroxyphenylpyruvate dioxygenase (HPD) plays an important role in the tyrosine catabolic pathway and as such in the management of tyrosine-related inherited metabolic disorders (TIMD), i.e. hereditary tyrosinemia (HT) type I and alkaptonuria (AKU). HPD is an iron-(II)-dependent, non-haem, oxygenase and catalyzes the conversion of 4-hydroxyphenylpyruvate (HPP) to homogentisate (HGA), which comprises the second step in the catabolism of tyrosine [1], [2], [3]. Inhibition of this key enzyme prevents the formation of HGA and following toxic intermediates, and as such alleviates the harmful phenotype of these TIMD [1,4] (Fig. 1). Such an inhibitor, that is used to treat TIMD, is nitisinone [2-(2-nitro-4-trifluoromethylbenzoyl—1,3-cyclohexadione; NTBC], a member of the β-triketone family of herbicides (i.e. mesotrione, sulcotrione, and tembotrione) but many other inhibitors are being explored that may pave the way to more TIMD treatment options.

**Fig. 2:**
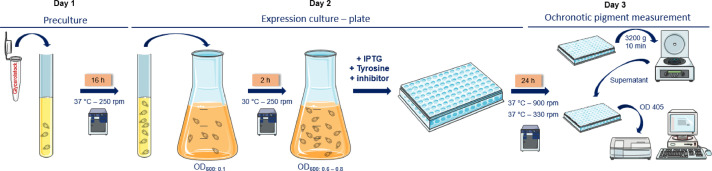
Schematic overview of the HTS assay

In search for new inhibitors of HPD, a screening system that allows to identify and evaluate the potency of new HPD inhibitors for the development of TIMD therapies is necessary. To fill this gap, we have developed a straightforward, colorimetric and non-laborious high-throughput screening (HTS) assay in bacteria which depends on the activity of recombinant expressed human HPD [1]. For this HTS, *Escherichia coli* is the preferred organism to use for the production of recombinant enzymes because genetic engineering as well as HTS setups in microtiter plates are well-established [5,6]. Furthermore, due to the natural presence of transaminases, *E. coli* is also capable of easily converting tyrosine to HPP, the first step in the metabolism of tyrosine, which is then further metabolized into HGA by the expressed human HPD enzyme. Subsequently, accumulated HGA will auto-oxidize to benzoquinone acetic acid due to the absence of HGD in *E. coli* and self-polymerize to produce a melanin-like ochronotic pigment. The melanin-like ochronotic pigment is also known as pyomelanin and exhibits a characteristic brown color. Our newly developed colorimetric bioassay is based on the quantification of pyomelanin, derived from tyrosine under aerobic and physiological conditions relevant to humans. In the presence of an HPD-inhibitor, this ochronosis process will be reduced or even prevented when HPD activity is blocked [7], [8], [9] (Fig. 2).

Other methods, using a coupled enzyme reaction to perform high-throughput screening have been published but these are not the most efficient. Compared to our HTS assay, coupled enzyme HTS systems are more laborious, time-consuming and complex [10], [11], [12], [13].

Altogether, the main purpose of this bacterial whole-cell HTS assay based on human HPD is to analyze the inhibitory capacity of new potent inhibitors of the human HPD enzyme and thereby to evaluate their therapeutic potential [1].

## Set-up

### Equipment

- Spectrophotometer (Eppendorf Biophotometer Plus).
- Microtiter plates (96-wells, v-shaped) for protein expression (Greiner Bio-One GmbH, Frickenhausen, Germany).
- Deep well microtiter plates (96-wells, v-shaped) for protein expression (VWR, Leuven, Belgium).
- Microtiter plates (96-wells, flat bottom) (Greiner Bio-One, Frickenhausen, Germany).
- Shaking incubator (Multitron II, Infors GmbH, Einsbach, Germany).
- Multitron II Infors Shaker (Infors AG, Bottmingen, Switzerland).
- Plate reader Tecan Sunrise® or Tecan Infinite® M200 pro (Tecan Group Ltd., Männedorf, Switzerland).
- Erlenmeyer culture flask (Carl Roth GmbH + Co. KG).
- Test tube (Carl Roth GmbH + Co. KG).
- Biophotometer (Eppendorf).
- Pipette (10 µL – 100 µL – 1000 µL) (Eppendorf).

### Stock solutions and reagents

- *Lysogeny Broth (LB) medium*: 5 g yeast extract (Sigma-Aldrich), 10 g peptone from casein, acid digest (Sigma-Aldrich) and 10 g sodium chloride (Sigma-Aldrich) are dissolved in 900 mL ultrapure water. Adjust the pH of the solution to 6.7–7.0 by use of 1 N sodium hydroxide or 1 N hydrochloric acid. Further, dilute the solution till 1000 mL with ultrapure water. Loosely close the cap of the bottle (otherwise the bottle may explode) and then cover the top of the bottle with aluminum foil. Sterilize by autoclaving at 121 °C for 20 min and prevent boil-over by using a bigger bottle. Screw the top of the bottle, allow the solution to cool down, label and store at room temperature.
- *LB medium + 1 % w/V D-glucose (LBG medium)*: 5 g yeast extract (Sigma-Aldrich), 10 g peptone from casein, acid digest (Sigma-Aldrich), 10 g sodium chloride (Sigma-Aldrich) and 10 g D-glucose (Sigma-Aldrich) are dissolved in 900 mL ultrapure water. Adjust the pH of the solution to 6.7–7.0 by use of 1 N sodium hydroxide or 1 N hydrochloric acid. Further dilute the solution till 1000 mL with ultrapure water. Loosely close the cap of the bottle (otherwise the bottle may explode) and then cover the top of the bottle with aluminum foil. Sterilize by autoclaving at 121 °C for 20 min and prevent boil-over by using a bigger bottle. Screw the top of the bottle, allow the solution to cool down, label and store at room temperature.
- *Kanamycin sulfate from Streptomyces kanamyceticus (*50 mg/mL*):* Kanamycin (Sigma-Aldrich) is dissolved in double distilled water as a 1:1000 solution according to the final concentration of 50 ng/mL in culture. The stock solution is sterilized by filtration with a 0.22 µm sterile filter and can be stored for 6 months at −20 °C. Briefly vortex before use.
- *Sodium-L-tyrosine (*75 mg/mL*):* Sodium-L-tyrosine (Sigma-Aldrich) is dissolved in double distilled water as a 1:100 solution according to the final concentration of 0.75 mg/mL in culture. The stock solution is sterilized by filtration with a 0.22 µm sterile filter and can be stored for 6 months at −20 °C.
- *Isopropyl-β-D-thiogalactopyranoside / IPTG (*500 mM*):* 1.1915 g IPTG (Sigma-Aldrich) is dissolved in 10.0 mL double distilled water to obtain a 500-fold IPTG stock solution according to the final concentration of 1 mM in culture. The stock solution is sterilized by filtration through a 0.22 µm sterile filter and can be stored for 6 months at −20 °C. Vortex before use.
- *NTBC (*3 mM or 1 mg/mL*):* 50 mg NTBC (Yecuris) and 300 mg NaHCO_3_ (Sigma-Aldrich) are dissolved in 50 mL double distilled water by use of a magnetic stirrer at 30–40 °C. Evaluate if the solution is completely dissolved and filter with a 0.22 µm sterile filter and aliquot the stock solution. Keep protected from light and store for a maximum of 6 months at −20 °C. Briefly vortex before use.
- *Mesotrione (*3 mM*):* 51 mg mesotrione (Sigma-Aldrich) and 300 mg NaHCO_3_ (Sigma-Aldrich) are dissolved in 50 mL double distilled water by use of a magnetic stirrer at 30–40 °C. Evaluate if the solution is completely dissolved and filter with a 0.22 µm sterile filter and aliquot the stock solution. Keep protected from light and store for a maximum of 6 months at −20 °C. Briefly vortex before use.
- *Sulcotrione (3 mM):* 49 mg sulcotrione (Sigma-Aldrich) and 300 mg NaHCO_3_ (Sigma-Aldrich) are dissolved in 50 mL double distilled water by use of a magnetic stirrer at 30–40 °C. Evaluate if the solution is completely dissolved and filter with a 0.22 µm sterile filter and aliquot the stock solution. Keep protected from light and store for a maximum of 6 months at −20 °C. Briefly vortex before use.
- *Tembotrione (*3 mM*):* 66 mg tembotrione (Sigma-Aldrich) and 300 mg NaHCO_3_ (Sigma-Aldrich) are dissolved in 50 mL double distilled water by use of a magnetic stirrer at 30–40 °C. Evaluate if the solution is completely dissolved and filter with a 0.22 µm sterile filter and aliquot the stock solution. Keep protected from light and store for a maximum of 6 months at −20 °C. Briefly vortex before use.
- 25% glycerol stock of *E. coli* C43 (DE3) strain (Lucigen Corporation, Middleton, WI, USA) containing pET42b(+) (Empty vector, EV) or pET42b(+)-HPD plasmid.

NOTE: The used C43 (DE3) contains the kanamycin resistant pET42b(+) plasmid vector to maintain selection pressure.

### General safety note

*E. coli* C43 (DE3) is classified as Biosafety Level 1 (BSL1), which means they pose a minimal potential threat to laboratory workers and do not cause infection or disease in healthy adults.

## Protocol

Before commencing any work, sterilize the workspace using 70% ethanol. Always use personal protective equipment!

### Inoculation and incubation of bacterial cells containing 4-hydroxyphenylpyruvate dioxygenase

The importance of a pre-culture is that cell growth should be monitored before induction and this can be accomplished by using LB medium, a nutrient-rich media commonly used to culture bacteria. Leaky expression of the toxic mRNA/protein, resulting in slower growth rate of the bacterial cells, can be avoided by the addition of 1% w/v glucose, i.e. a carbon source [5].

#### Inoculation and growth of the preculture

- Add 6 mL LBG to a test tube and add kanamycin to a final concentration of 50 µg/mL.
- From the frozen glycerol stock (25%) of *E. coli* C43 (DE3) – EV and C43 (DE3) – HPD, a pinch is taken by means of a sterile pipette tip and dropped into the liquid LBG + antibiotic. Swirl the tube and close with a non-airtight cap.
- The preculture is incubated and grown overnight (18 ± 2 h) at 250 rotations per minute (rpm) and 37 °C in a shaking incubator.

### Colorimetric bioassay high-throughput screening

After overnight inoculation (18 ± 2 h), the preculture is diluted in fresh medium as it allows the cells to return to their logarithmic growth phase whereafter they reach an optimal cell density for induction of protein expression [14]. As soon as HPD expression is induced, HPP will be converted to HGA which will result in the formation of ochronotic pigment and the medium will turn brown. In case you have a potent inhibitor of HPD, the medium remains yellowish at a certain concentration.

#### Inoculation and growth of the expression culture

- After overnight incubation, check the tubes for bacterial cell growth, which is characterized by a cloudy media.
- Measure the OD_600_ of the overnight culture by means of a cuvette to determine if the cells are at the appropriate growth stage. Fill one cuvette with 1000 µL of sterile LBG medium and use it as a blank by pressing the ‘blank’ button on the Biophotometer. Fill another cuvette with 900 µL of sterile LBG medium and add a 100 µL aliquot of the overnight preculture. Pipette up and down until it is homogeneous. Measure by pressing the ‘sample’ button on the Biophotometer. Take into account the 10-fold dilution.

NOTE: Ensure that the overnight culture is well mixed and that the cells are not settled before taking an aliquot. Use clean cuvettes and insert it in the proper orientation in the Biophotometer.

- Use the overnight culture to inoculate a volume of LB (+ add kanamycin to a final concentration of 50 µg/mL) in an Erlenmeyer culture flask to an OD_600_ = 0.1 by using following formula:

$a=\;\frac{0.1\;x\;b}{OD_{600}}$ (1)
a: volume (mL) of overnight culture necessary to obtain OD_600_ = 0.1 in LB medium
0.1: Desired OD_600_
b: volume of LB medium (mL) (200 µL per well * 96 wells per plate * # plates)
OD_600_: measured OD_600_ of preculture, considering the 10-fold dilution
NOTE: a fill of 1/5 of nominal flask capacity should not be exceeded for microbial cultures, e.g. 20 mL for a 100 mL Erlenmeyer flask, to ensure shaking as aeration and oxygen and nutrient availability are made possible by shaking. This time LB is used, and not LBG, as glucose represses protein expression!

- Swirl the culture flask and close with a non-airtight cap.
- Incubate the cultures at 250 rpm, 70% R.H. and 30°C in a shaking incubator.
- Monitor the OD_600_ of the cultures every half hour by measuring the OD by means of a cuvette. Fill one cuvette with 1000 µL of sterile LB medium and use it as a blank by pressing the ‘blank’ button. Fill another cuvette with 1000 µL aliquot of expression culture. Measure by pressing the ‘sample’ button on the Biophotometer. No dilution must be considered.
- When OD_600_ reaches 0.6–0.8, the mid-log phase, protein expression can be induced by addition of a proper inducer such as the lactose analog isopropyl-β-D-thiogalactopyranoside or IPTG [5]. Add IPTG to a final concentration of 1 mM in the Erlenmeyer flask containing the expression culture.

NOTE: At the mid-log phase the bacteria are in the exponential growth phase which means they express chaperones that help to remove misfolded proteins and will reduce formation of inclusion bodies. If you induce too early, the protein yield will be reduced and/or bacterial growth will be affected.

**Fig. 3:**
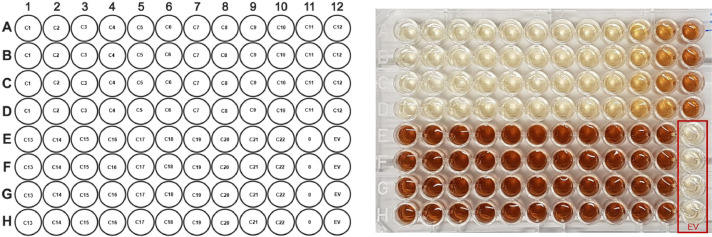
**Left panel/ Example of plate layout:** 22 different concentrations of the inhibitor can be tested on a 96-multi (deep) well plate with 4 replicates per concentration to obtain a dose-response inhibition curve. **Right panel/ Result of 96-multiwell expression plate 24 h after induction:** 22 different concentrations of nitisinone are tested. Formation of brown ochronotic pigment was observed 24 h after protein induction. The addition of nitisinone decreased pyomelanin formation in a dose-dependent manner with increasing inhibitor concentration.
- Pour the expression culture in a sterile reagent reservoir and add 200 µL (normal well format) / 600 µL (deep well format) of the induced culture to each well of the 96-(deep) well V-bottom expression MTP using a multichannel pipette. If the cell culture rests too long in the reservoir, pipette the culture gently up and down by using the multichannel pipette.
- Close the 96-well plate with a sterile lid and seal with tape.
- Incubate for 24 h in a shaking incubator at 900 rpm (normal well format) / 330 rpm (deep well format), 37 °C and 70% R.H.

- Add sodium-L-tyrosine to a final concentration of 0.75 mg/mL in the expression culture in the Erlenmeyer flask.
- Mix the contents by swirling the Erlenmeyer flask.
- In the meantime, make a dilution series of the desired inhibitor starting from the stock solution (e.g. 3 mM NTBC) in different Eppendorf tubes (1.5 mL) to obtain a final concentration of:
  ○ 10 µM (C1): 20 µL 3 mM INHIBITOR + 180 µL ddH_2_O
  ○ 9 µM (C2): 18 µL 3 mM INHIBITOR + 182 µL ddH_2_O
  ○ 8 µM (C3): 16 µL 3 mM INHIBITOR + 184 µL ddH_2_O
  ○ 7 µM (C4): 14 µL 3 mM INHIBITOR + 186 µL ddH_2_O
  ○ 6 µM (C5): 12µL 3 mM INHIBITOR + 188 µL ddH_2_O
  ○ 5 µM (C6): 10 µL 3 mM INHIBITOR + 190 µL ddH_2_O
  ○ 4 µM (C7): 8 µL 3 mM INHIBITOR + 192 µL ddH_2_O
  ○ 3 µM (C8): 6 µL 3 mM INHIBITOR + 194 µL ddH_2_O
  ○ 2 µM (C9): 4 µL 3 mM INHIBITOR + 196 µL ddH_2_O
  ○ 1 µM (C10): 4 µL 3 mM INHIBITOR + 396 µL ddH_2_O
  ○ 0.9 µM (C11): 1.8 µL 3 mM INHIBITOR + 198.2 µL ddH_2_O
  ○ 0.8 µM (C12): 1.6 µL 3 mM INHIBITOR + 198.4 µL ddH_2_O
  ○ 0.7 µM (C13): 1.4 µL 3 mM INHIBITOR + 198.6 µL ddH_2_O
  ○ 0.6 µM (C14): 1.2 µL 3 mM INHIBITOR + 198.8 µL ddH_2_O
  ○ 0.5 µM (C15): 1.0 µL 3 mM INHIBITOR + 199 µL ddH_2_O
  ○ 0.4 µM (C16): 80 µL 30 µM INHIBITOR + 120 µL ddH_2_O
  ○ 0.3 µM (C17): 60 µL 30 µM INHIBITOR + 140 µL ddH_2_O
  ○ 0.2 µM (C18): 40 µL 30 µM INHIBITOR + 160 µL ddH_2_O
  ○ 0.1 µM (C19): 20 µL 30 µM INHIBITOR + 180 µL ddH_2_O
  ○ 0.075 µM (C20): 15 µL 30 µM INHIBITOR + 185 µL ddH_2_O
  ○ 0.05 µM (C21): 10 µL 30 µM INHIBITOR + 190 µL ddH_2_O
  ○ 0.01 µM (C22): 2 µL 30 µM INHIBITOR + 198 µL ddH_2_O
- Vortex each Eppendorf tube before use.
- Add 6.9 µL (normal well format)/20 µL (deep well format) of an inhibitor dilution solution to each well of the 96-well V-bottom expression MTP by means of multi-dispensing pipette, with 4 replicates per concentration (for 0 µM and EV add 6.9 µL / 20 µL ddH_2_O) (Fig. 3):

### Spectroscopic quantification of ochronotic pigment

Depending on the potency of the inhibitor and its concentration, the formation of the ochronotic pigment can be visually seen after 24 h as a brown colored medium.

- Turn on the centrifuge and cool down till 4 °C.
- After 24 h, remove the 96-(deep) well V-bottom MTP expression plate from the shaking incubator.
- Put the 96-(deep) well V-bottom MTP expression plate in the centrifuge. In case you have an odd number of MTP plates make sure the centrifuge is in balance by filling an empty plate with water.

NOTE: Weigh the 96-(deep) well V-bottom MTP expression plate on a precision balance. Weigh an empty 96-(deep) well V-bottom MTP plate with lid on the same precision balance and fill it with water by means of a Pasteur pipette until it has the same weight as the 96-(deep) well V-bottom MTP expression plate.

- Centrifuge the plates at 3200 g during 10 min at 4 °C. Make sure the centrifuge is balanced!
- Remove the plates from the centrifuge and discard the tape and lid.
- Transfer 150 µL supernatant of the 96-(deep) well V-bottom MTP expression plate into a 96-well flat bottom MTP plate by means of a multichannel pipette. Do NOT touch the pellet with the pipette tip. Use new tips per column! Discard the 96-(deep) well V-bottom MTP expression plate after the supernatant is transferred.
- Turn the Tecan microplate reader on and select the Tecan I control Software on the computer. Define the used 96-well plate (i.e. Greiner 96 Flat Transparent).
  ○ Select the wells to be measured. By default, all the wells are already selected. If you want to select other wells click and drag across the plate layout.
  ○ Select at the ‘Measurements’ menu ‘Absorbance’.
  ○ **Wavelength**: Measurement: 405 nm. Turn off ‘reference’ reading.
  ○ **Read**: ‘number of flashes’: default. Do not use ‘settle time’ parameter (0 ms).
  ○ **Multiple reads per well**: do not select as you have a homogeneous solution.
  ○ Movements (shaking) and temperature is not required.
- Open the plate reader at ‘Instruments → Movements → Plate out‘ and put the 96-well flat bottom MTP plate into the holder. Close the plate reader at ‘Instruments → Movements → Plate in‘.
- Start measurement by pressing ‘Start’. Data will be directly driven into Excel in a matrix format.
- Remove your plate at ‘Instruments → Movements → Plate out‘ and discard the 96-well flat bottom MTP plate from the holder. Close the plate reader at ‘Instruments → Movements → Plate in‘. Discard the 96-well flat bottom plate.
- Save your data.

### Analysis of the results

- Calculate the normalized OD_405_ (OD_405-N_) with following equation:

$OD_{405-N}=\;OD_{405}-\;OD_{405-EV}$ (2)
OD_405_: measured OD_405_ of the sample
OD_405-EV_: mean OD_405_ of the EV

- Calculate the activity percentage (Act. %) with following equation:

$Act.\%=(OD_{405-N}/OD_{405-M})*100\;\%$ (3)

OD_405-M_: mean of OD_405_ measured at 0 µM of inhibitor (i.e. 100% activity)

**Fig. 4:**
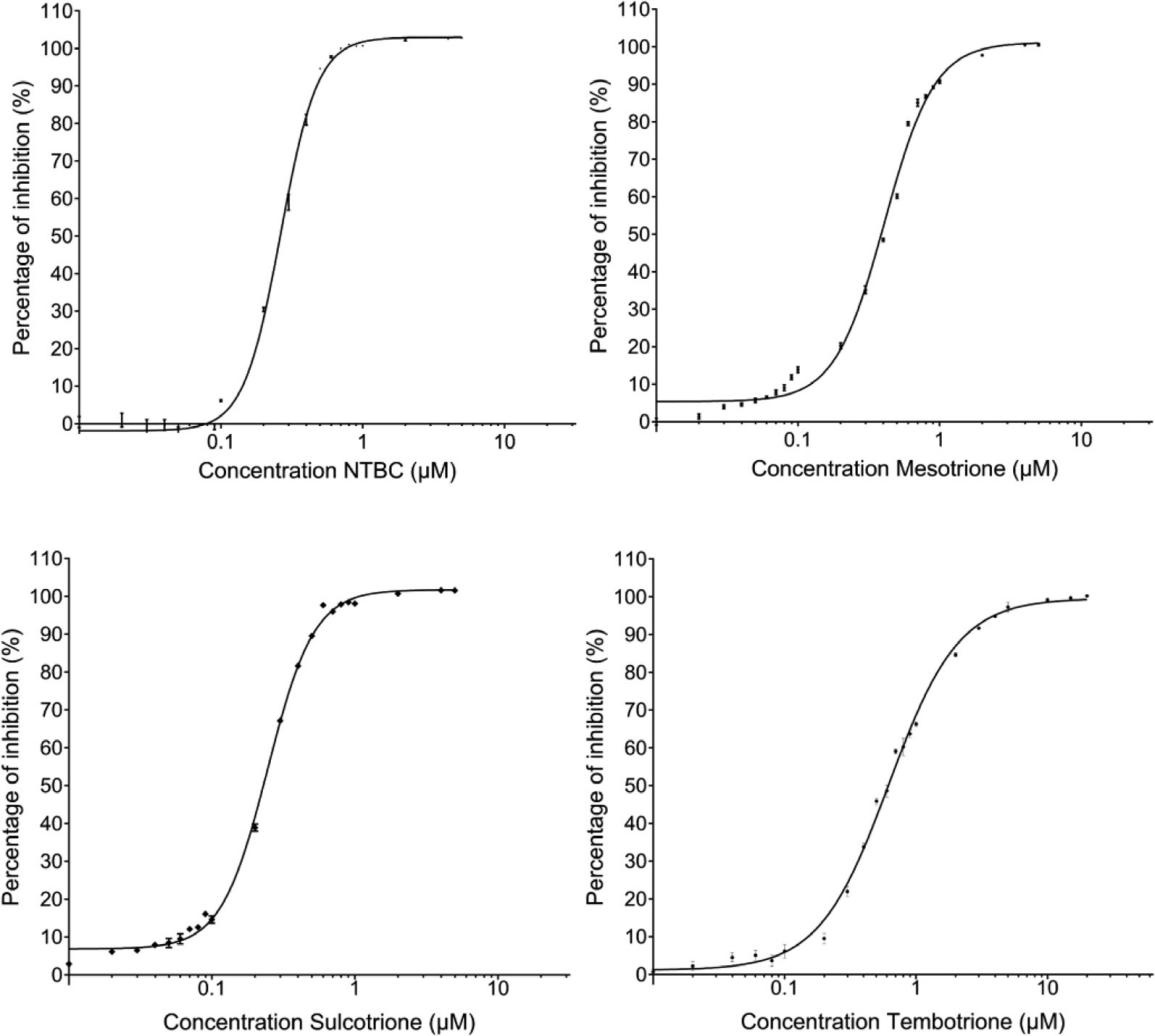
**The percentage of inhibition (PIN)** was calculated following equation (2), (3) and (4). The calibration curve for nitisinone, mesotrione, sulcotrione and tembotrione was fitted by sigmoidal logistic four-parameter equations.

- Calculate the percentage of inhibition (PIN) with following equation (Fig. 4):

PIN = 100 – Act. % (4)

**Supplementary material *and/or* Additional information**: Human HPD is an important drug target that is involved in HT1, however, a simple and robust HTS assay is lacking in drug discovery. As such, we have developed an HTS colorimetric whole-cell bioassay that can be used as a first and fast screening and especially for labs with limited funding. Many advantages are related to this assay. It is a simple, cheap and fast assay as the induction time of the recombinant HPD enzyme is only 24 h where other assays have induction times of 48 or even 72 h [7]. To allow high-throughput screening, the bioassay was adapted to the 96-well microplate format. Previously reported 96-well plate format assays are based on the coupled enzyme reaction of the isolated HPD enzyme with the crude extract of HGD and the formation of maleylacetoacetate, measured at 318 nm. This is also a reliable assay, however, it is laborious, time-consuming and there is a need for addition of exogenous co-factors what makes it more expensive [11], [12], [13],[15], [16], [17], [18]. Another advantage of this whole-cell bioassay is that you can use L-tyrosine as a substrate instead of the much more expensive HPP due to the natural presence of tyrosine aminotransferases in *E. coli* [19], [20], [21], [22]*.* Furthermore, we can conclude that we have developed a very robust HTS assay that was rigorously validated both for pharmacological relevance, as well as for robustness of assay performance according to the NCGC guidelines [1]. This validation consists of a 3-day Plate Uniformity study and a Replicate-Experiment study, and, in addition, signal variability and spatial uniformity were assessed. Moreover, this HTS assay has been executed in two different labs, independent from each other, without undergoing any substantive changes. The plate uniformity and signal variability results, in 96-well microplate format, are represented in Table 1 and were used to calculate the CVs and Z′ factor values, taking into account 1 replicate per inhibitor or inhibitor concentration, in order to represent a real-life HTS assay. Maximum signal plate CVs were 1.82–4.06%, midpoint signal CVs were 2.74–7.56%, and minimum signal CVs were 2.28–5.86%. These CVs all pass the <20% criterion. Plate Z′ values were 0.87 ± 0.03 whilst the recommended acceptance criterion is Z′ factor ≥0.40 and related to the screening it can be seen as an excellent and robust assay. The inter-plate tests, all within-day fold shifts <2 and all average (between-) day fold shifts <2 also meet the criteria [1,23].

**Table 1:** Results of the (intra) plate uniformity study. H, high signal; M; medium signal; L, low signal: CV, coefficient of variation; Mid. %, normalised mid signal; SW, signal window; Z′, Z′ factor. All max signal and all mid signal (unnormalised) CVs are <20%. All normalised mid signal (mid. %) SD's <20, SW's type="Other">2 and Z′ factors >0.4. All min (low) SD's < Min (max (High) SD, mid SD). Z′ and CV values were calculated taking into account 1 replicate per compound or concentration, thereby mimicking a real-life HTS assay.

Day	Plate	Type	Mean	SD	CV	Mid%	SW	Z'
1	1	H	2.38	0.07	3.12	61.25 ± 2.49	23.75	0.88
		M	1.15	0.05	4.36			
		L	0.37	0.01	2.28			
	2	H	2.15	0.06	2.76	70.11 ± 3.02	26.14	0.88
		M	0.91	0.05	5.82			
		L	0.39	0.01	3.01			
	3	H	2.04	0.04	1.82	85.13 ± 1.78	40.49	0.91
		M	0.64	0.03	4.62			
		L	0.39	0.01	2.51			
2	1	H	2.20	0.06	2.96	68.86 ± 3.94	23.90	0.87
		M	0.97	0.07	7.30			
		L	0.41	0.01	3.55			
	2	H	2.32	0.06	2.41	80.89 ± 2.66	30.12	0.89
		M	0.78	0.06	6.43			
		L	0.42	0.01	3.41			
	3	H	2.16	0.07	3.19	71.77 ± 3.93	21.59	0.85
		M	0.91	0.07	7.56			
		L	0.41	0.02	3.94			
3	1	H	2.05	0.07	3.20	78.74 ± 2.83	21.36	0.86
		M	0.76	0.05	6.06			
		L	0.42	0.01	3.03			
	2	H	2.25	0.05	2.40	64.13 ± 1.57	30.50	0.89
		M	1.06	0.03	2.74			
		L	0.40	0.02	3.87			
	3	H	2.12	0.09	4.06	73.96 ± 3.00	16.00	0.81
		M	0.86	0.05	5.98			
		L	0.41	0.02	5.86			

Due to evaporation during the incubation periods or plate stacking, plate edge or side effects can occur in HTS assays and as such spatial uniformity needs to be examined. Plates that do not exhibit material edge or drift effects are the overall requirement. Row and column drift were assessed using the high and medium signals from left-to-right and top-to-bottom. No drift effects that exceed 20% were observed, i.e. maximum drift observed for the high signal and medium signal was 6.23% and 13.79% resp., so material drift effects can be excluded [1]. We foresee that the throughput and robustness can even be further improved by miniaturizing the assay to 384 and 1536 well-format using adjusted equipment including automatic pipetting systems and liquid-handling robotics.

There is one major concern involving variable absorption effects of different drugs in *E. coli*, causing incomparable inhibitory effects as knowledge of the influx and efflux transporters for xenobiotic, present in bacteria, is remarkably limited. However, a recent study revealed that, besides the diffusion of xenobiotics across whatever phospholipid bilayer, many transporters interact with xenobiotics [24]. If *E. coli* cells are capable of taking up large antibiotic molecules, we assume that the absorption (and absorption rate) of small chemical molecules, i.e. triketone inhibitors and derivatives, is not affected. Furthermore, the aim of our HTS system is not to establish therapeutical concentrations of each inhibitory compound. This assay is used as a first and fast HTS system to screen a large library of different inhibitors of human HPD in the search of possible hits.

## Declaration of Competing Interest

The authors declare that they have no known competing financial interests or personal relationships that could have appeared to influence the work reported in this paper.
